# Genome-wide analysis of the transcription factor binding preference of human bi-directional promoters and functional annotation of related gene pairs

**DOI:** 10.1186/1752-0509-5-S1-S2

**Published:** 2011-05-04

**Authors:** Bingchuan Liu, Jiajia Chen, Bairong Shen

**Affiliations:** 1 Center for Systems Biology, Soochow University, Suzhou, 215006, China; 2 School of life Science and Technology, Tongji University, Shanghai, 200092, China; 3 School of Chemistry and Biological Engineering, Suzhou University of Science and Technology, Suzhou, 215009, China

## Abstract

**Background:**

Bi-directional gene pairs have received considerable attention for their prevalence in vertebrate genomes. However, their biological relevance and exact regulatory mechanism remain less understood. To study the inner properties of this gene organization and the difference between bi- and uni-directional genes, we conducted a genome-wide investigation in terms of their sequence composition, functional association and regulatory motif discovery.

**Results:**

We identified 1210 bi-directional gene pairs based on the GRCh37 assembly data, accounting for 11.6% of all the human genes owning RNAs. CpG islands were detected in 98.42% of bi-directional promoters and 61.07% of unidirectional promoters. Functional enrichment analysis in GO and GeneGO both revealed that bi-directional genes tend to be associated with housekeeping functions in metabolism pathways and nuclear processes, and 46.84% of the pair members are involved in the same biological function. By fold-enrichment analysis, we characterized 73 and 43 putative transcription factor binding sites(TFBS) that preferentially occur in bi-directional promoters from TRANSFAC and JASPAR database respectively. By text mining, some of them were verified by individual experiments and several novel binding motifs were also identified.

**Conclusions:**

Bi-directional promoters feature a significant enrichment of CpG-islands as well as a high GC content. We provided insight into the function constraints of bi-directional genes and found that paired genes are biased toward functional similarities. We hypothesized that the functional association underlies the co-expression of bi-directional genes. Furthermore, we proposed a set of putative regulatory motifs in the bi-directional promoters for further experimental studies to investigate transcriptional regulation of bi-directional genes.

## Background

According to the orientation and status of the 5’ end, the adjacently located genes can be arranged in convergent, divergent or tandem configuration[1]. Among these categories, the divergent gene arrangement is found more frequently than expected by chance in the human genome, accounting for about 10% of all human genes[2,3]. Bi-directional gene pair is defined as two genes arranged in a head-to-head (adjacent 5’ ends) fashion on opposite strands of DNA with less than 1,000 bp between their transcription start sites(TSS)[1]. Accordingly, the entire intervening region between the two TSSs is designated as a putative bi-directional promoter. A gene is termed as uni-directional if no oppositely oriented TSS is found within 10 kb upstream of the given TSS, or if a similarly oriented TSS is found at least 1 kb upstream. Thus the entire 1 kb of 5’ flanking DNA is considered as the uni-directional promoter.

Considerable attention has been focused on bi-directional genes in recent years. Examples including LRRC49/THAP10[4], SURF-1/SURF-2[5], COL4A1/COL4A2[6], PCD10/SERPINI1[7] and HAND2/DEIN[8] have been identified in human through individual experiments. A considerable number of bi-directional gene pairs were found to be conserved among mammalian species[9,10]. Since evolutionary conservation usually indicates functional implications, we proposed that bi-directional gene organization is under selection to fulfil a specific functional role. Whereas most of the bi-directional gene pairs have been found in the process of studying a single gene, a genome-wide analysis of their function and physiologic consequences is currently insufficient.

The expression data obtained from biotechnologies such as SAGE and microarray indicated a correlated expression profile between bi-directional genes[11-13]. Based on the assumption that ‘co-expression implies co-regulation’, the requirement for co-regulation of functionally related genes appears to underlie the observed co-expression. However, it is still under discussion whether the co-expression evolved merely as a consequence of their physical proximity or if function dictated their co-regulation. There are several examples of bi-directional gene pairs that are related by function, e.g. in DNA repair[1,2], aging[14], *de novo* purine synthesis[15] and carcinogenesis[5]. Despite this observation, a systematic study on the degree of internal co-function of the bi-directional genes has not been carried out to date.

More recent studies have suggested an intrinsic difference in nucleotide composition of bi-directional promoters compared to uni-directional ones[1,2,13,16]. These characteristic feature lead us to hypothesize that divergent genes will be transcribed with a special set of regulatory signals. Currently our understanding of transcription regulation relies greatly on experimental identification of prospective regulatory regions. Yet many specifics underlying the regulatory design are unknown. Therefore, it seems necessary to re-evaluate the underlying mechanisms and biological relevance of bi-directional promoters systematically.

**Figure 1:**
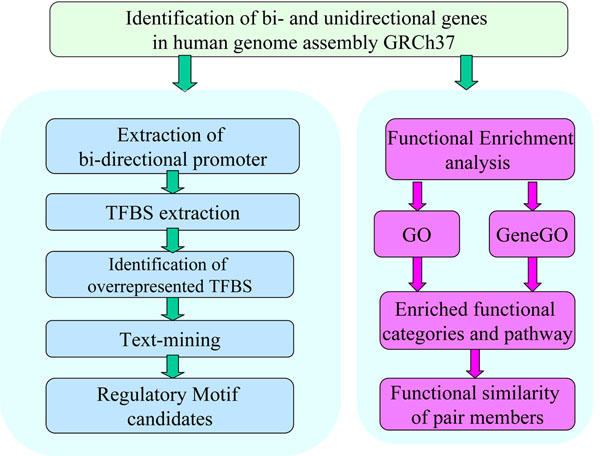
Diagrammatic Representation of the Research Scheme

In the present study, we have undertaken a genome-wide survey of gene organization in the human genome. To reveal functions collectively performed by such bi-directional genes, we mapped them to the Gene Ontology (GO) and GeneGO pathways. We also explored the functional similarity between the genes on the plus strand and those on the minus strand. We devoted our effort into exploring the binding preference of transcription factors on the bi-directional promoters and statistically identified a set of over-represented transcription factor binding sites(TFBS) in bi-directional promoters, the research scheme is shown in figure 1.

## Results

### Identification of bi-directional and uni-directional genes

We calculated the distances between the transcription start sites of nearest gene neighbors for four kinds of combination of the clusters and the result was showed in table 1. We identified 1210 bi-directional gene pairs based on the curated transcript cluster NMs and NRs, accounting for 11.67% of all the genes owning RNAs, which agrees the view that bidirectional gene pairs are prevalent in the human genome. If only transcripts with conclusive mRNA were reserved, 878 bi-directional gene pairs, in the proportion of 9.31%, were discovered upon the removal of pairs consisting of NMs and NRs. Redundant gene pair entries that share the same intergenic sequence were removed to yield 822 bi-directional gene pairs for the analyses.

**Table 1:** Distribution of bi-directional gene pairs on each chromosome

Chromosome	Total Gene Number	Chromosome Length(bp)	all	NR+NM	NM	NR
1	4165	249,250,621	129	97	85	2
2	2858	243,199,373	99	74	66	1
3	2210	198,022,430	76	58	52	0
4	1750	191,154,276	40	36	27	0
5	1930	180,915,260	69	51	45	0
6	2836	171,115,067	87	68	61	1
7	2408	159,138,663	65	50	34	1
8	1677	146,364,022	47	32	27	0
9	1849	141,213,431	61	48	37	2
10	1672	135,534,747	49	33	27	0
11	2468	135,006,516	81	66	58	0
12	2051	133,851,895	65	52	45	0
13	851	115,169,878	19	12	11	0
14	1793	107,349,540	62	54	47	0
15	1512	102,531,392	33	25	21	0
16	1706	90,354,753	83	68	54	1
17	2239	81,195,210	105	86	73	2
18	710	78,077,248	18	12	9	1
19	2388	59,128,983	87	72	61	0
20	1063	63,025,520	29	25	22	1
21	545	48,129,895	19	15	8	3
22	1075	51,304,566	33	28	19	1
X	2118	155,270,560	47	32	28	0
Y	491	59,373,566	5	0	0	0
sum			1408	1094	917	16

### CpG islands are preferentially located in bi-directional promoters

**Figure 2:**
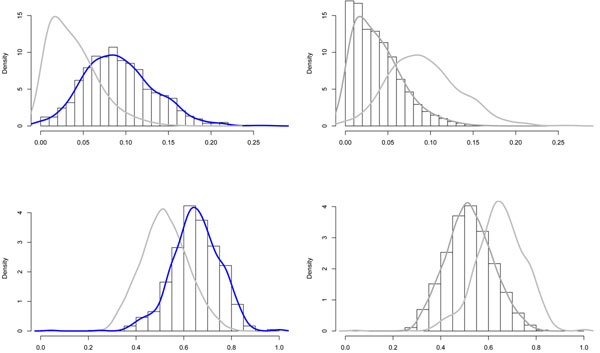
**Density Distribution of CpG Islands between Bi-directional Genes and Unidirectional Genes(The figure is reproduced with permission from the rights owner Liu,B.**[28])

There have been contradictory observations on the CpG island frequency in bi-directional promoters. Adachi *et al.*[1,16] considered the presence of CpG island to be a common feature of bidirectional promoters. In contrast, Takai, *et al*. [13] reported that CpG islands are not preferentially associated with bidirectional promoters. The author attributed the discrepancy to the different criteria adopted to define a CpG island. In order to rationalize these controversial observations, we performed genome-wide computational analysis of the bi-directional promoters on the basis of two different definition systems. According to traditional definition by Gardiner-Garden[17], CpG islands were detected in 809 bi-directional promoters, representing 98.42% of a total of 822 pairs. A lower percentage of 61.07% was recorded for unidirectional promoters. Based on more strict criteria[18] (DNA fragment no less than 500 bp with GC-content >= 55% and Obs/Exp value >=0.60), CpG-islands were present in 86.37% of bidirectional promoters compared to 28.48% of uni-directional promoters. In addition, we analyzed pure IG sequence to remove the difference caused by the extended IG region. Invariably the frequency of CpG island in bi-directional promoters is higher than those in unidirectional ones. As shown in Figure 2, the CpG density in bidirectional promoters (histogram in top left) is significantly higher than that in unidirectional promoter (histogram in top right) in all comparisons. Consistent with a significant enrichment of CpG-islands, bidirectional promoters feature a high C+G content.

### Functional Enrichment of Bi-directional Genes

#### Gene ontology associated with bi-directional promoter regulation

Genes regulated by bi-directional promoters were examined for functional classifications and associations. Among the 1,644 genes involved in the 822 human bi-directional gene pairs, 1,121, 1,219, and 1,256 genes were directly annotated by ‘biological process’, ‘molecular function’ and ‘cellular component’ subcategories in GO annotation system, respectively. We found several GO classes significantly over-represented among bi-directional genes. Cellular, metabolic and biosynthetic processes emerged as the most significantly enriched functional class. GO items of cell cycle and its child nodes were also significantly presented. Cellular response to stress or stimulus and their related subclasses of damage response, break repair were also focused. To summarize, the most enriched GO categories correspond to the known physiological roles of the cell, indicating that bi-directional genes are frequently involved in basic cellular metabolic processes. See Additional file 1 for the complete list of enriched GO terms.

#### Functional similarities among the paired genes

**Figure 3:**
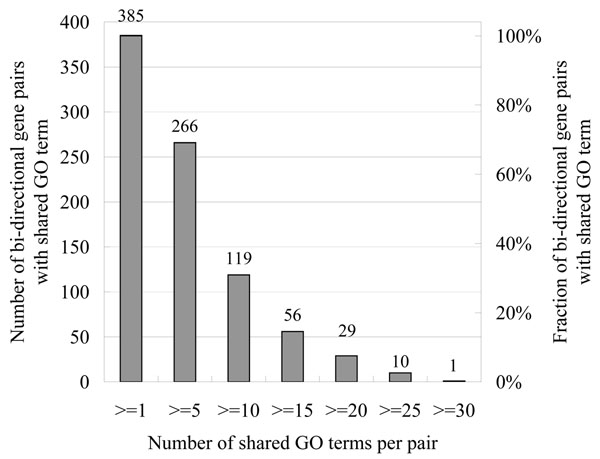
**Number and percentage of gene pairs that overlapped for various GO terms** The X-axis represented the number of shared GO terms per gene pair. Number and percentage of gene pairs that overlapped by various GO terms among the total 385 pairs are plotted on the Y-axis.

Among 822 annotated bi-directional gene pairs, we found 385 pairs (46.84%) whose plus and minus genes share at least one GO term, with SARS2/MRPS12 having most GO terms in common. Number and percentage of gene pairs that overlapped for various GO terms were showed in Figure 3. (See Additional file 2 for detailed list of shared GO terms). Such shared or related function supports the hypothesis that bi-directional genes are more likely to be functionally associated than uni-directional genes.

**Figure 4:**
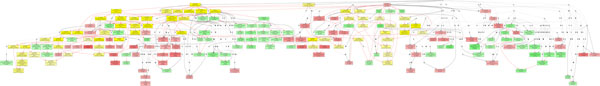
**The shared GO terms and P value in Biological Process subcategory** Red boxes represent GO terms that are occupied exclusively by genes on the plus strand; Green boxes represent GO terms that are occupied exclusively by genes on the minus strand, while yellow ones were common terms shared by plus and minus genes within the bi-directional gene pair. The color darkens with the significance of enrichment.

We also provided separate estimates for each of the Gene Ontologies. We obtained 337 annotated pairs in subcategory "cellular component", 185 pairs in "molecular function" and 146 pairs in "biological process" respectively. It’s observed that, in general, bi-directional gene products are more likely to perform coordinated roles in the same cellular component, compared to the other two subsystems. Figure 4 illustrated the shared GO terms and P values in subcategory "cellular component".

Then we set out to find out the GO terms that represent coordinated functions of bi-directional pairs. In Biological Process, the GO terms related to metabolic process and its branch such as primary metabolic process, cellular process and biopolymer biosynthetic process topped the list of both gene pair members. Their child nodes were focused on RNA (mRNA, ncRNA) metabolic process, cellular (macromolecule or biopolymer) catabolic process, organelle organization, mitotic cell cycle *etc.* In molecular function, the GO terms involved in DNA-directed RNA polymerase activity, RNA methyltransferase activity, purine NTP-dependent helicase activity, NAD or NADH binding, NADH dehydrogenase (quinone) activity, *etc.* are significantly over-represented as compared to others. In Cellular Component, we found that bi-directional genes tend to be tightly associated into the same class of organelle, organelle envelope, nucleus, nucleoplasm, nucleolus, membrane-bounded or non-membrane-bounded organelle, *etc.* Interestingly, almost all the items shared by the two divergent genes are related to metabolism and energy transfer. We proposed that genes involved in functions including metabolism, are more likely to be organized in the head-to-head configuration.

#### GeneGO pathway enrichment

Based on the P values from MetaCore™, totally we found 45 pathways that are significantly enriched with bi-directional genes out of the total 451 distinct pathways. According to the different classification criterion, the 45 pathways were assigned to 18 regulatory processes, 8 protein function, 4 disease maps and 15 metabolic maps. Extreme enrichment occurred for, in order of descending significance level, NHEJ mechanisms of DSBs repair, Oxidative phosphorylation, Nucleotide excision repair and GTP-XTP metabolism, Chromosome condensation in prometaphase, Role of Brca1 and Brca2 in DNA repair. Enriched pathways are further clustered into larger functional categories according to the GeneGO annotation. Regulatory processes/Cell cycle and Regulatory processes/DNA-damage ranked among the top enriched functional categories. Table 2 lists some most enriched categories ordered in decreasing level of significance.

**Table 2:** Statistically enriched GeneGO Pathway categories

Pathway category	*P*-value
Regulatory processes/Cell cycle	5.35E-09
Regulatory processes/DNA-damage	2.07E-08
Metabolic maps/Metabolic maps (common pathways)/Energy metabolism	1.12E-06
Metabolic maps/Metabolic maps (common pathways)	1.03E-04
Metabolic maps/Metabolic maps (common pathways)/Nucleotide metabolism	6.96E-04
Metabolic maps/Metabolic maps (common pathways)/Vitamin and cofactor metabolism	5.71E-03

#### Functional enrichment in GeneGO versus GO

So far we have been analyzing the level of gene function enrichment using two function annotation schemes respectively. The GO results show a clear agreement with those derived from the GeneGO pathways. For example, the GO terms that are significantly enriched include genes that are engaged in processes such as DNA metabolic process, which correspond to the Metabolic maps/Metabolic maps (common pathways)/Nucleotide metabolism pathway in GeneGO; Cell cycle, which corresponds to the same pathways in GeneGO; response to DNA damage stimulus, which corresponds to Regulatory processes/DNA-damage in GeneGO. This agreement is also apparent in that ‘‘DNA repair’’ is the most enriched GO term and ‘‘* DNA damage_Nucleotide excision repair,’’ which corresponds to the Regulatory processes/DNA-damage pathway, is one of the top enriched pathways in GeneGO as well.

### Bi-directional promoters are characterized by a distinct collection of putative transcription factor binding sites

**Figure 5:**
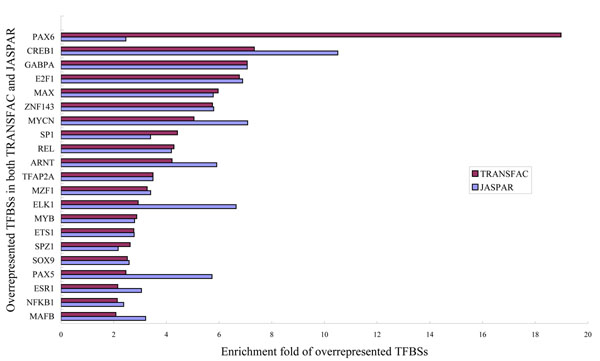
The over-represented TFBSs that overlapped in TRANSFAC and JASPAR

We characterized the enrichment of known motifs from TRANSFAC and JASPAR in bi-directional promoters relative to background uni-directional promoters. Based on the Jaspar PSSM information, we categorized 43 transcription factors as over-represented and 6 as under-represented. In the TRANSFAC database, 73 TFBSs found increased presence in bi-directional promoters. Complete lists of over-represented motifs and their enrichment folds are provided as Additional file 3. Although there is slight difference between the two databases, a large majority of the TFBSs overlap. The overlapped TFBSs and their over-represented folds were illustrated in Figure 5. We hypothesize that over-represented motifs correspond to transcription factors that are more likely to bind to bidirectional promoters than to unidirectional promoters. In contrast, under-represented motifs correspond to transcription factors that preferentially regulate unidirectional promoters. Shared motifs show no preference.

We further investigated the experimental evidence supporting the roles of these transcriptional factors in regulating certain bi-directional genes. Table 3 lists the experimentally validated TFBS that occurred in bi-directional promoters. Some of the reported physiological functions are consistent with our functional enrichment analysis. For example, previous work[19] has demonstrated that GABPA regulates genes involved in a variety of cellular processes including adipocyte differentiation, mitochondrial respiration, and neuromuscular signaling, corresponding to enriched GO terms of cell cycle, cellular and metabolic processes and their child nodes. E2F1 are observed to regulate cell growth during the G0/G1-S phase transition, and over-expression of E2F1 induces apoptosis and DNA synthesis in quiescent fibroblasts[20]. These are in agreement with the significantly enriched GeneGO pathways such as Regulatory processes/Cell cycle and Regulatory processes/DNA-damage.

**Table 3:** The experimentally validated TFBS that occurred in bi-directional promoters

TF name	Fold Enrichment	Regulated gene pair	Reference
GABPA	7.069	Gapba/Atp5j PREPL-C2ORF34	[19] [29]
E2F1	6.893	TK/KF genes	[10]
NFY	5.255	Mrps 12/Sarsm PREPL-C2ORF34 Mrps 12/Sars2	[30] [29] [31]
SP1	3.398	OSGEP/APEX Gapba/Atp5j DEIN/HAND2 HSF-1/Bop1 E14/ATM	[32] [19] [8] [33] [34]
CCAAT box	2.687	BRCA1/NBR2 GPAT/AIRC OSGEP/APEX mOsgep/mApex	[35] [36] [37] [38]
NF1	2.591	Pxmp2/Polel	[39]

Interestingly, the over-represented recognition sequence for MYC, ELK1, NF-Y, SP1, ATF, GABPA, SREBP-1, NF-E2, STAT5A, NF-1 as well as SOX-9 rank among the most conserved motifs found in human promoters[21].

Given the enrichment of these motifs in bi-directional promoters and their strong evolutionary conservation across mammalian promoters, we assume that the predicted TFBSs located within bi-directional promoters are more likely to be functional in co-regulation than other TFBSs. Interestingly, it would appear that TFs within the same family tend to have similar binding preference. A TFBS is either over-represented or under-represented in parallel with other family members. These observations suggest a common mode of expression across the family members of transcription factors.

## Discussion

In this study, 11.6% of the human genes were shown to be arranged in a head-to-head fashion, and this proportion is slightly larger than most of the previous report[2], except that Piontkivska *et al.*[3] reported a number of 1,369 bi-directional promoters. The inconsistency was partly due to the update of TSS coordinates during the accumulation of EST and mRNA evidence. In addition, we used the much more highly curated RefGene track instead of the spliced human ESTs collection, because the large and complicated ESTs data containing thousands of transcripts captured by oligo-capping techniques will lead to an overestimation of the frequency of transcripts, and then introduce false positive result. What’s more, our work focus on the pure mRNA gene pairs and a large part of non-coding RNA, transcribed RNA and miscRNA are excluded from further analysis. Herein we provided a solid evidence for the previous observation[1] that bi-directional promoters had a significant enrichment of CpG-islands as well as a high GC content. Since CpG island is usually the targets of regulation by methylation, it may induce changes in chromatin structure that can confer either positive or negative effects on transcription. Misregulation of bi-directional promoters elicited by mutation or hypermethylation will simultaneously silence genes on both sides. Loss of their vital biological function well explains the role bi-directional genes in the development of human diseases such as aging[14], brain disease[7] and oncogenesis[4].

Our study provided insight into the function constraints of bi-directional genes. Functional enrichment analysis in GO and GeneGO both revealed that bi-directional genes are often associated with housekeeping functions. GO terms, including metabolic process, such as DNA, RNA, biopolymer or macromolecule metabolism, as well as nuclear processes, such as DNA repair and replication or cell cycle regulation are significantly enriched. The GeneGO pathways that are involved in growth or proliferation, such as those engaged in Energy metabolism, Nucleotide metabolism, Vitamin and cofactor metabolism, tend to be more enriched with bi-directional genes. Pathways in genetic information processing (transcription, translation and DNA repair) and cell cycle tend to be enriched as well. To summarize, bi-directional genes are significantly enriched in housekeeping functions such as metabolism pathways and nuclear processes.

Further analyses revealed that the significant functional categories are more likely to be shared by bi-directional genes. This indicated that the bi-directional genes are strongly biased toward functional similarities and coordinated regulation. We postulate that for bi-directional genes involved in basic biological processes, coordinated regulation ensures their synchronized action and thus minimizes transcriptional error. In contrast, genes with less coordinated regulation may be involved in pathways that are more flexible in responding to environmental changes.

We compared the TFBSs between bi- and uni-directional promoters according to their rate of occurrence. We discovered several transcription factors that preferentially regulate bi-directional promoters. Some of the TFBSs matched well with experimentally determined ones and several novel binding motifs were also identified. These bi-directional gene associated motifs may be envisaged as the best candidates for functional regulatory elements. In addition, the motif search result could help identify novel genes, which is linked to a known gene via a bi-directional promoter. And these genes probably perform important conserved functions.

We are also aware of some limitations in our analysis. The motifs for the identification of TFBSs are still incomplete, and the evolutionary importance of the over-representation of TFBS remains to be elucidated. Although some of their function are indicated by functional categories (GO terms) of experimental verified motifs, conclusive evidence of the role played by regulatory factors in the co-regulation of the two genes will be tested in experiments. Eventually, the combination of computational and experimental approaches will permit us to construct mechanistic models of regulatory transcription networks of bi-directional genes. It would be interesting, as a future endeavor, to examine these regulatory elements in other species in a similar fashion and compare the results to those obtained herein. Comparative analyses of these regulators across multiple species will validate our predictions by their appearance in another species. A related work is still in progress.

## Conclusions

In this work, we conducted a systematic investigation of bi-directional gene organization focusing on sequence features, functional association and regulatory motif discovery. We confirmed known properties of bi-directional gene organization and also provided new observations. We found that bi-directional gene pairs show a higher probability to be functionally associated, formulating hypotheses that the requirement for co-regulation of functionally related genes is a possible cause for the observed co-expression of bi-directional genes. We also proposed that a special set of motifs in the bi-directional promoters play a role in transcriptional regulation of bi-directional genes. Our data also provide the putative regulatory motifs for experimental studies to investigate how the expression of divergent gene pairs is regulated.

## Methods

### Identification of bi-directional and uni-directional genes in human genome

Human genome assembly GRCh37, released as NCBI Build36 and Ensemble release 55, was downloaded from Genome Reference Consortium (ftp://ftp.ncbi.nlm.nih.gov/genbank/genomes/Eukaryotes/vertebrates_mammals/Homo_sapiens/GRCh37/Primary_Assembly/assembled_chromosomes/FASTA/). Gene annotation (NCBI Build36) was retrieved from the NCBI Entrez Gene ftp site (ftp://ftp.ncbi.nlm.nih.gov/gene/DATA/). The transcript annotation including transcription orientation, strand, starting site (hg19) was downloaded from hg19 RefGene table from UCSC Genome Browser (http://hgdownload.cse.ucsc.edu/goldenPath/hg19/database/). A total of 45,408 genes (excluding mitochondrial genome) and 31,357 transcripts were collected and filtered for redundancy. This resulted in 44,293 non-redundant items of RefSeqs transcripts. Genes without clear mRNA information (NR, XR and XM) were filtered to ensure the exact transcription of all the genes. The 28520 mRNAs were collapsed into 21757 unique and non-overlapping clusters, which were further ranked according to their chromosome position and TSS coordinates to determine the adjacent gene pairs. Discrimination of bi-directional gene pairs and uni-directional genes was performed by a perl script according to the definition by Trinklein. *et al*[2]. Redundant gene pair entries that share the same intergenic sequence were removed.

### Extraction of bi-directional promoter region

Based on the mapping information of gene and its transcripts, possible multiple TSSs were assessed. The intergenic regions between bi-directional genes’ TSS were taken as bi-directional promoters. For uni-directional genes the region of 1000 bp upstream of the TSS were extracted as promoter. Promoter regions were extracted from the chromosome fasta files of the latest GRCh37 version genome assembly datasets. (ftp://ftp.ncbi.nlm.nih.gov/genbank/genomes/Eukaryotes/verte brates_mammals/Homo_sapiens/GRCh37/Primary_Assembly/ assembled_chromosomes/FASTA/).

### Analysis of Promoter Sequences

The intergenic sequences of bidirectional genes were extended in both sides symmetrically into 1000 bp to meet the definition of a CpG island length. CpG island finder script[22] was run with two types of parameter criteria, %GC>=50, Obs/Exp>=0.60, length 500 and %GC >=55, Obs/Exp>=0.60, length 500 respectively. CpG frequency within the bi-directional and uni-bidirectional promoters was calculated.

### Evaluation of Functional Enrichment

We utilized Gene Ontology (GO) categories (http://www.geneontology.org/) and a commercial software MetaCore-GeneGO Pathway Maps (http://www.genego.com/metacore.php) to group functionally related genes and to contrast the functional distribution of bi-directional genes to the average distribution in the whole genome. The analysis of over-represented GO terms for bi-directional genes was performed by the GOEAST[23]. Statistical enrichment of a category was quantified using the Hypergeometric test method. Yekutieli multi-test adjustment method was applied to correct for multiple testing.

Genes were then mapped to GeneGO database by MetaCore™ tools to infer pathways preferentially targeted by bi-directional genes. In MetaCore™, the statistical significance of the enriched pathways is indicated by a *P* value yielded from the Fisher’s exact test. The False discovery rate (FDR) is also applied to correct for multiple testing.

### Discovery of over-represented transcription factor binding sites

Putative TFBS in promoter regions were searched for matches to the position-weight matrix(PWM) in the JASPAR[24,25] and TRANSFAC[26] database. Predetermined PWMs for 73 and 87 vertebrate TFBSs were extracted from TRANSFAC(public version 7.0) and JASPAR PSSM, respectively. Alignment of PWMs on genomic sequence was performed with COTRASIF[27] (http://biomed.org.ua/COTRASIF/). TFBSs within bi-directional promoters were categorized as over-represented, shared or under-represented at 2-fold threshold. Over-represented TFBS was defined as whose normalized number of binding sites in bidirectional promoters is 2-fold larger than those in unidirectional ones while under-represented means the normalized number of binding sites in bidirectional promoters is 2-fold smaller than the number of sites in a single unidirectional promoter. Shared motif is the intermediate state. A total of 18840 uni-directional promoters was used to give a contrast of bi-directional genes.

## Competing interests

The authors declare that they have no competing interests.

## Authors' contributions

BL carried out the promoter compositional analysis, participated in the TFBS categorization and drafted the manuscript. JC participated in the functional enrichment analysis, performed the statistical analysis and draft the manuscript. BS conceived of the study, and participated in its design and coordination. All authors read and approved the final manuscript.

## Supplementary Material

Additional file 1 TableS1 Identification and statistics of GO terms enriched with bi-directional genes. Click here for file

Additional file 2 TableS2 Functional similarities for plus and minus genes within annotated bi-directional gene pairs. Click here for file

Additional file 3 TableS3 The enrichment of TFBS in bi-directional promoters relative to uni-directional promoters. Click here for file
